# Measurement of Ocular Compliance Using iPerfusion

**DOI:** 10.3389/fbioe.2019.00276

**Published:** 2019-10-25

**Authors:** Joseph M. Sherwood, Elizabeth M. Boazak, Andrew J. Feola, Kim Parker, C. Ross Ethier, Darryl R. Overby

**Affiliations:** ^1^Department of Bioengineering, Imperial College London, London, United Kingdom; ^2^Wallace H. Coulter Department of Biomedical Engineering, Georgia Institute of Technology and Emory University, Atlanta, GA, United States; ^3^Atlanta VA Medical Center, Atlanta, GA, United States; ^4^George W. Woodruff School of Mechanical Engineering, Georgia Institute of Technology, Atlanta, GA, United States

**Keywords:** sclera, cornea, ocular rigidity, ocular compliance, glaucoma

## Abstract

The pressure-volume relationship of the eye is determined by the biomechanical properties of the corneoscleral shell and is classically characterised by Friedenwald's coefficient of ocular rigidity or, alternatively, by the ocular compliance (OC), defined as dV/dP. OC is important in any situation where the volume (V) or pressure (P) of the eye is perturbed, as occurs during several physiological and pathological processes. However, accurately measuring OC is challenging, particularly in rodents. We measured OC in 24 untreated enucleated eyes from 12 C57BL/6 mice using the iPerfusion system to apply controlled pressure steps, whilst measuring the time-varying flow rate into the eye. Pressure and flow data were analysed by a “Discrete Volume” (integrating the flow trace) and “Step Response” method (fitting an analytical solution to the pressure trace). OC evaluated at 13 mmHg was similar between the two methods (Step Response, 41 [37, 46] vs. Discrete Volume, 42 [37, 48] nl/mmHg; mean [95% CI]), although the Step Response Method yielded tighter confidence bounds on individual eyes. OC was tightly correlated between contralateral eyes (*R*^2^ = 0.75, *p* = 0.0003). Following treatment with the cross-linking agent genipin, OC decreased by 40 [33, 47]% (*p* = 0.0001; *N* = 6, Step Response Method). Measuring OC provides a powerful tool to assess corneoscleral biomechanics in mice and other species.

## Introduction

The eye is a deformable, pressurised globe with an outer layer composed of soft connective tissues known as the corneoscleral shell. The pressure-volume (P-V) relationship of the eye is determined by the biomechanical properties of the corneoscleral shell and is important in a number of contexts. Specifically, in any situation where the volume of the eye is perturbed, there will be a resulting change in pressure, the magnitude of which depends on this P-V relationship. Such perturbations occur when delivering therapeutic agents directly into the eye (Wen et al., 2017), when measuring intraocular pressure (IOP) (Gloster, 1965), and during cardiac-induced ocular blood volume pulsations (Coleman and Trokel, 1969). Similar effects occur during laboratory measurements of the physiological parameters controlling IOP, such as outflow facility (Li et al., 2016; Stockslager et al., 2016; Reina-Torres et al., 2017). Additionally, the biomechanical properties of the corneoscleral shell may themselves be important in several ocular pathologies, including glaucoma, myopia, and keratoconus. Thus, accurate measurement of the P-V relationship of the eye is valuable.

Classically, the *P* − *V* relationship of the eye is characterised by Friedenwald's coefficient of ocular rigidity, *K*, which links relative changes in intraocular pressure, *P*, to relative changes in intraocular volume, *V* according to Gloster (1965):

(1) $$\begin{array}{l} \frac{dP}{P}=k\frac{dV}{V}=KdV \end{array}$$

The value of *K* = *k*/*V* is typically assumed to be constant because most perturbations have a negligible effect on *V*, although *K* itself (and hence *k*, as *V* changes very little) has been reported to be pressure-dependent (Silver and Geyer, 2000).

An alternative parameter to describe the *P* − *V* relationship of the eye is “ocular compliance,” ϕ = *dV*/*dP*. The advantage of ϕ is that ocular compliance directly describes an absolute change in volume per unit change in pressure, as opposed to *K* that describes a relative change in pressure per unit change in volume. Thus, ϕ is exactly equivalent with a compliance element used in lumped parameter modelling, such as the Windkessel model commonly used in cardiovascular mechanics. Lumped parameter modelling provides a valuable tool to isolate the dynamic mechanical response of the eye from that of the measurement system (see section Accounting for the Dynamic Response of the Measurement System).

Measurements of ocular compliance have typically analysed the pressure spike in response to a bolus injection of fluid into the eye, as performed in mice (Lei et al., 2011), rats (Ficarrotta et al., 2018), and tree shrews (Stockslager et al., 2016). This approach is similar to measurement of *K* in humans and eyes from larger species (Pallikaris et al., 2005). However, as highlighted recently (Campbell et al., 2018), the dynamic response of the measurement system when coupled to the eye, and the pressure dependence of ocular compliance itself, make it difficult to correctly measure ocular compliance, particularly in eyes of smaller animals, which are more amenable for research. How to accurately and robustly measure ocular compliance therefore remains an open question.

Rodents are useful models for investigating ocular biomechanics, due in part to their genetic malleability and ready availability of rodent-specific tools and reagents, in addition to offering considerable ethical and practical benefits over primates. However, their diminutive size makes experimental measurements challenging. Motivated by an analogous challenge to accurately measure outflow facility (the hydraulic conductance of the primary fluid drainage pathway in the eye) in mice, we developed *iPerfusion* (Sherwood et al., 2016), which uses open-loop pressure control, rather than the more traditional closed-loop control with a flow source (Overby et al., 2002; Lei et al., 2011; Ko et al., 2014). This approach has increased the speed and accuracy of outflow facility measurements and has revealed novel aspects of outflow physiology (Sherwood et al., 2016; Madekurozwa et al., 2017).

We here report how we have extended the application of the *iPerfusion* system to measure ocular compliance. We also describe the development of complementary protocols and analytical methods necessary for precise and accurate measurement of ocular compliance in mice. With relatively minor modifications, these protocols, and methods could be adapted to other species.

## Theoretical Formulation

### Ocular Compliance

The pressure-volume relationship of the corneoscleral shell may be described in terms of the ocular compliance, defined as

(2) $$\begin{array}{l} \phi =\frac{dV_{\phi }}{dP}\approx \frac{V_{\phi }}{P} \end{array}$$

which represents the change in intraocular volume, *V*_ϕ_, per unit change in intraocular pressure, *P*. ϕ may also be interpreted as the slope of the volume-pressure relationship of the eye, where a smaller slope represents a less compliant, or stiffer, corneoscleral shell. Ocular compliance itself depends strongly on IOP, as seen for the case of the Friedenwald model (Equation 1) where ϕ = 1/(*KP*).

Alternatives to Friedenwald's model to describe the *P* − *V* relationship of the eye can be derived from first principles (McEwen and St. Helen, 1965; Woo et al., 1972; Collins and van der Werff, 2013). For example, by using Laplace's law and a Fung constitutive formulation for a collagenous corneoscleral shell of the form σ = *A*(*e*^αε^ − 1) (Fung, 1967) and by assuming small strains and uniform mechanical properties, an expression for the ocular compliance can be obtained as

(3) $$\begin{array}{l} \phi =\phi_{r}\left(\frac{P_{r,\phi }+\gamma }{P+\gamma }\right) \end{array}$$

where ϕ_*r*_ is defined as a reference compliance that applies at a reference pressure of *P*_*r*,ϕ_ [see (Ethier et al., 2004) and Supplementary Material S1]. *P*_*r*,ϕ_ can be selected as desired, but the physiological value of IOP is a natural choice. In the derivation of Equation (3) (Supplementary Material S1), the term γ is a function of the material properties and geometry of the eye. However, in this study, γ is treated as an empirical parameter that is determined experimentally. For the special case of γ = 0 (Equation 3) reduces to the Friedenwald model, ϕ = ϕ_*r*_(*P*_*r*,ϕ_/*P*), where *K* = 1/(ϕ_*r*_*P*_*r*,ϕ_).

### Accounting for the Dynamic Response of the Measurement System

Any determination of ocular compliance requires that the eye be interfaced with a measurement system, which will inevitably have its own compliances and flow resistances. The interaction of this system with the eye will cause the measured pressures and/or flow rates to differ from the true intraocular pressure or the true flow rate entering the eye. Thus, the behaviour of the measurement system itself must be considered in the measurement of ocular compliance.

There are two approaches to measure ocular compliance: (i) by causing a change in ocular volume and measuring the resulting change in IOP (the *applied volume approach*), or (ii) by causing a change in IOP and measuring the resulting change in ocular volume (the *applied pressure approach*). We previously described the applied volume approach (Campbell et al., 2018). Here, we focus on the applied pressure approach, which has the advantage of a faster temporal response compared to the applied volume approach and thus increases experimental throughput.

To aid understanding of the derivation and application of the methods described in sections Theoretical Formulation and Methods, we provide a schematic figure (Figure S1) and a nomenclature of the key variables in the analysis (Supplementary Material S2).

#### Experimental Setup

**Figure 1:**
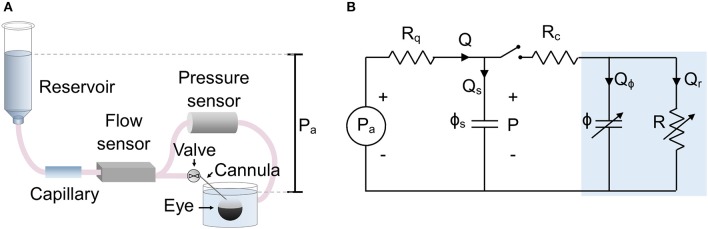
The setup used to measure ocular compliance. **(A)** Schematic of the experimental setup, showing relevant components. **(B)** Lumped parameter (equivalent circuit) model of the setup, with blue shaded region indicating the eye. Parameters are defined as follows: *P*_*a*_, applied pressure; *P*, measured pressure; *Q*, measured flow rate; *Q*_*s*_, flow rate into the compliance of the system; *Q*_ϕ_, flow rate into the compliance of the eye; *Q*_*r*_, flow through the aqueous humour outflow pathway of the eye; *R*_*q*_ = 1/*C*_*q*_, combined hydraulic resistance (inverse of hydraulic conductance, *C*_*q*_) of the flow sensor and capillary in series; *R*_*c*_, hydraulic resistance of the cannula; *R* = 1/*C*, hydraulic resistance of the outflow pathway (inverse of outflow facility, *C*); ϕ_*s*_, compliance of the measurement system; ϕ, compliance of the eye. Note that ϕ and *R* are strongly pressure-dependent.

The experimental setup is shown in Figure 1A, which is a modified form of the existing *iPerfusion* system previously developed for measuring outflow facility (Sherwood et al., 2016). An enucleated eye is fully immersed in a bath of isotonic saline and cannulated via the anterior chamber. Upstream of the cannula is an adjustable height reservoir mounted on a linear actuator (L35, Nanotec, Germany) to control the applied pressure, *P*_*a*_, measured with respect to the height of the fluid in the eye bath^1^. A thermal flow sensor (SLG150, Sensirion AG, Switzerland) measures the flow rate, *Q*, from the reservoir, and a differential pressure sensor (PX409, Omegadyne, US) measures the pressure upstream of the cannula relative to the eye bath, *P*. A glass capillary (inner diameter 100 μm, length 80 mm) located upstream of the flow sensor is added to increase hydraulic resistance and slow the system time response, thereby improving the sampling of flow and pressure data necessary to precisely measure ocular compliance. The perfusion system upstream of the valve (Figure 1A) is filled with water containing 0.02% (w/v) sodium azide to suppress biofilm formation. The tubing and cannula downstream of the valve and in contact with the eye, is filled with perfusate, typically “DBG”: sterile-filtered phosphate buffered saline (PBS) containing 5.5 mM glucose.

A lumped parameter model of the experimental setup is shown in Figure 1B. The applied upstream pressure, *P*_*a*_, is controlled by the actuated reservoir. *R*_*q*_ represents the sum of the hydraulic resistances of the capillary and flow sensor. ϕ_*s*_ is the system compliance, with contributions from the tubing and pressure transducer. *R*_*c*_ represents the hydraulic resistance of the cannula, and *R* is the hydraulic resistance of the aqueous humour outflow pathway (see below). ϕ is the ocular compliance. Note that ϕ and *R* are pressure-dependent. By the conservation of mass, the flow rate measured by the flow sensor, *Q*, can be written as the sum *Q* = *Q*_*s*_ + *Q*_ϕ_ + *Q*_*r*_, where *Q*_*s*_ is the flow rate into the system compliance, *Q*_ϕ_ is the flow rate into the ocular compliance, and *Q*_*r*_ is the flow rate through the aqueous humour outflow pathway.

Typical values are *R*_*q*_ = 4.5 *mmHg*/(μ*l*/min) and *R*_*c*_ = 0.01 – 0.5 *mmHg*/(μ*l*/min), with outflow resistance being approximately *R* = 200 *mmHg*/(μ*l*/min). To simplify the model, the resistance of the cannula is neglected, as *R*_*c*_ is much less than either *R*_*q*_ or *R*, and the pressure drop across *R*_*c*_ is much smaller than the typical step size in *P*_*a*_. Thus, ϕ_*s*_ and ϕ can be considered as parallel compliances, and the pressure reading *P* is equivalent to IOP.

#### Outflow Resistance

For an enucleated mouse eye, the reciprocal of the hydraulic resistance of the outflow pathways, known as the *outflow facility*, can be described by Sherwood et al. (2016)

(4) $$\begin{array}{l} C=\frac{1}{R}=C_{r}{\left(\frac{P}{P_{r,c}}\right)}^{\beta } \end{array}$$

where *C*_*r*_ is the outflow facility at a reference pressure *P*_*r,c*_, typically taken to be 8 mmHg to correspond to the physiological pressure drop across the outflow pathway. The non-linearity parameter β accounts for changing outflow facility with increasing pressure, as may occur for example due to anterior chamber deepening (Boussommier-Calleja et al., 2015; Sherwood et al., 2016).

### Measuring Ocular Compliance

To measure ocular compliance, we applied a series of pressure steps to the eye by elevating the adjustable height reservoir and determined the resulting pressure and flow rate into the eye for each pressure step. We here present and evaluate two methods of analysing the resulting data to calculate ocular compliance: (i) the *Discrete Volume Method*, in which the fluid volume entering the eye is calculated by integrating the flow rate signal over time, and (ii) the *Step Response Method*, in which the dynamic response of the recorded pressure trace is compared to an analytical solution using non-linear regression. Both methods aim to determine the parameters ϕ_*r*_ and γ by fitting Equation (3) to acquired values of the pressure and compliance calculated for each step. Due to the non-linearity of Equation (3), this requires iterative procedures, as described below.

#### Measuring Ocular Compliance Using the Discrete Volume Method

Using the discrete form of Equation (2), ocular compliance can be calculated as the change in intraocular fluid volume, *V*_ϕ_, for a given change in intraocular pressure, i.e., ϕ = *V*_ϕ_/*P*. However, ϕ itself is a function of pressure, *P* (Equation 3), and hence ϕ changes throughout the pressure step. It is therefore necessary to calculate, for each pressure step, both ϕ and the pressure to which this value of ϕ corresponds, termed *P*_ϕ_.

In response to a step increase in applied pressure, intraocular pressure increases from *P*_*j*−1_ at *t* = 0 to *P*_*j*_ = *P*_*j*−1_ + Δ*P*_*j*_ at *t* = *T*, where subscripts are an index for the pressure step. During this step, the intraocular volume changes by an amount $\int_{0}^{T}Q_{\phi }\;dt$. The ocular compliance measured during the *j*^*th*^ step can therefore be written as

(5) $$\begin{array}{l} \;\phi {|}_{P_{\phi ,j}}=\frac{1}{{\Delta P}_{j}}\int_{0}^{T}Q_{\phi }\;dt \end{array}$$

where Δ*P*_*j*_ = *P*_*j*_ − *P*_*j*−1_. Note that in Equation (5), ϕ is evaluated at *P*_ϕ, *j*_ (rather than *P*_*j*_) to indicate the pressure *P* that is consistent with the measured value of ocular compliance in Equation (3). By conservation of mass, *Q*_ϕ_ = *Q* − *Q*_*s*_ − *Q*_*r*_, and Equation (5) can be written as

(6) $$\begin{array}{l} \;\phi {|}_{P_{\phi ,j}}=\frac{1}{{\Delta P}_{j}}\int_{0}^{T}\left(Q-C\;P\right)\;dt\;-\;\phi_{s} \end{array}$$

since *Q*_*s*_ = ϕ_*s*_(*dP*/*dt*) and *Q*_*r*_ = *C P* (note that *C* depends on *P* and hence *t*, Equation 4). Equation (6) provides a relationship to determine the ocular compliance for the *j*^*th*^ step based on *Q* and *P* measured over that step. The calculated value of ocular compliance corresponds to a specific intraocular pressure *P*_ϕ, *j*_, which lies between *P*_*j*−1_ and *P*_*j*_. Using the mean value theorem and Equations (3) and (5), we obtain (see Supplementary Material S3):

(7) $$\begin{array}{l} P_{\phi ,j}=\frac{{\Delta P}_{j}}{\text{ln }\left(1+\frac{{\Delta P}_{j}}{P_{j-1}+\gamma }\right)}-\gamma \end{array}$$

where γ is defined in section Ocular Compliance.

#### Measuring Ocular Compliance Using the Step Response Method

The step response method compares the transient response in the measured pressure, *P*, to an analytical solution in order to estimate ocular compliance. Perturbation analysis was used to determine the step response of the system in which both the ocular compliance and outflow resistance are pressure-dependent (see Supplementary Material S4). First order analysis, i.e., omission of terms of order ${\left({\Delta P}_{j}/P_{j}\right)}^{2}$ or higher, yields

(8) $$\begin{array}{l} P=P_{j}\left(1-\frac{\Delta P}{P_{j}}\left(\frac{1}{\lambda_{j}+\left(1-\lambda_{j}\right)e^{t/\tau_{j}\;}}\right)\right) \end{array}$$

where Δ*P*_*j*_ = *P*_*j*_ − *P*_*j*−1_ represents the step change in intraocular pressure measured between two sequential steps. The time constant for the *j*^*th*^ step is given by

(9) $$\begin{array}{l} \tau_{j}=\frac{\phi_{j}+\phi_{s}}{C_{q}+C_{j}\left(1+\beta \right)} \end{array}$$

and the parameter λ_*j*_ is given by

(10) $$\begin{array}{l} \lambda_{j}=\frac{\Delta P_{j}}{P_{j}}\left(\frac{1}{\phi_{j}+\phi_{s}}\right)\left(\frac{\beta {\left(\beta +1\right)C}_{j}\tau_{j}}{2}+\left(\frac{P_{j}\phi_{j}}{P_{j}+\gamma }\right)\right) \end{array}$$

The subscript *j* on pressure, facility, ocular compliance and λ indicates their steady state values at the end of the *j*^*th*^ pressure step (i.e., ϕ_*j*_ is the ocular compliance evaluated at *P* = *P*_*j*_).

## Methods

### Protocol to Measure Ocular Compliance

The experimental protocol to measure ocular compliance is described in detail below and is summarised in Figure S1. Sampling of all flow rate *Q* and pressure *P* signals was performed at 1,000 Hz and down-sampled to 20 Hz by averaging data within 50 ms non-overlapping windows. Each step response was analysed using both the Discrete Volume and Step Response Methods.

#### Measurement of the System Compliance

The system compliance ϕ_*s*_ was measured prior to cannulating the eye by closing the valve located immediately upstream of the cannula and applying four sequential pressure steps by raising the reservoir from 5 to 25 mmHg in increments of 5 mmHg. Since the valve was closed, all of the flow measured by the flow sensor entered the system compliance (*Q* = *Q*_*s*_). Hence, Equation (6) reduces to

(11) $$\begin{array}{l} \phi_{s,j}=\frac{1}{{\Delta P}_{j}}\int_{0}^{T}Q\;dt \end{array}$$

The integral was calculated numerically for each step using the trapezoidal rule with the period *T* of each step equal to 5 min. ϕ_*s,j*_ values did not vary with pressure, hence ϕ_*s*_ was defined as the average of the four measurements. For the present experiments, measured values of ϕ_*s*_ ranged from 2.5 to 10.6 *nl*/*mmHg*.

#### Measurement of the Step Response of the Eye

Eyes were submerged in a temperature-controlled bath (35°C) and mounted in a custom-designed holder that supported the eye during the cannulation without the need for glue, which would affect the apparent ocular compliance. Eyes were cannulated via the anterior chamber using pulled glass micropipettes with an outer diameter of 75–120 μm and a bevel angle of 60°. During the cannulation, the applied pressure was set to 8 mmHg, followed by a 30 min acclimatisation period at 8 mmHg to allow the eye to equilibrate to the perfusion environment. After acclimatisation, a variety of pressure stepping protocols were used. Typically, we started at 5.5 *mmHg* applied pressure and increased to 23 *mmHg*, in steps of 2.5 *mmHg*. Following each step, the flow rate was considered to have stabilised when a straight line fit to flow rate vs. time over a 5 min moving window yielded a slope that was <3 nl/min/min continuously for 1 min. *Q*_*j*_ and *P*_*j*_ were then calculated by applying a 1st order Savitsky-Golay filter with a frame length of 1 min to the last 4 min of data from each step, and then taking the average of the filtered data.

#### Removal of Errant Steps

Occasionally, we measured a poor quality response to an applied pressure step (“errant step”) and removed the data for that step. Typical reasons for removal of a step include a sudden change in flow rate, which is attributable to debris becoming lodged or dislodged within the cannula, or a prolonged transient response or “drift,” which is attributable to progressive deepening of the anterior chamber that increases outflow facility. To identify steps with irregular transient responses, we applied two objective criteria. First, we removed any steps that required longer than 10 min to stabilise, based on the criterion described in section Measurement of the Step Response of the Eye. Second, we removed any steps that did not conform to the transient response predicted by the analytical solution:

(12) $$\begin{array}{l} Q^{*}=\frac{Q-Q_{j}}{Q_{\text{max}}-Q_{j}}=\frac{1}{\lambda +\left(1-\lambda \right)e^{t/\tau \;}} \end{array}$$

where Q^*^ is a normalised flow rate, *Q*_*j*_ is the stable value of *Q* at the end of the step (as defined in section Measurement of the Step Response of the Eye), and *Q*_max_ is the measured flow rate at *t* = 0. Q^*^ data were filtered using a 1st order Savitsky-Golay filter with a frame length of 10 s. Non-linear regression was used to calculate the best fit of Equation (12) to the filtered data, referred to as $Q_{fit}^{*},$ where λ and τ were assigned to be free parameters. An individual step was eliminated if the 95th percentile of the absolute residuals from the quantity $|Q_{fit}^{*}-Q^{*}|\left(Q_{\max}-Q_{j}\right)$ exceeded an empirically defined threshold of 15 nl/min. Figure S2 shows example flow traces from regular and errant steps, and a demonstration of the advantages of the omitting errant data. Of 237 total steps included in the current study, 6 were removed based on the criteria above.

#### Calculation of Ocular Compliance Using the Discrete Volume Method

Having determined ϕ_*s*_ and removed irregular steps, all that remains to calculate the ocular compliance for the *j*^*th*^ step using Equation (6) is to account for the pressure-dependence of outflow facility, *C*. In principle, this could be done by incorporating Equation (4) into Equation (6). However, deviations of the actual measured values of outflow facility at each step (*C*_*j*_ = *Q*_*j*_/*P*_*j*_) from the idealised form given by Equation (4) cause the integrand in Equation (6) to trend toward a non-zero value, such that the integral does not converge over the duration of the step. This can be avoided by using a linear interpolation of outflow facility based on the actual measurements of flow and pressure according to

(13) $$\begin{array}{l} C=C_{j-1}+\left(\frac{P-P_{j-1}}{{\Delta P}_{j}}\right)\left(C_{j}-C_{j-1}\right) \end{array}$$

To determine *P*_ϕ,*j*_, we apply Equation (7). However, as γ is unknown, we perform an iterative procedure using data from all steps to estimate γ and the value of *P*_ϕ,*j*_ for each step. Starting with the first order approximation that *P*_ϕ,*j*_ ≈ *P*_*j*_ − Δ*P*_*j*_/2 (see Supplementary Material S3, Equation S8), we use non-linear regression to fit Equation (3) to the measured values of ϕ_*j*_ (obtained from Equation 6) assigning *P* = *P*_ϕ,*j*_ for each step. This yields an estimate of γ (and ϕ_*r*_) that we then use to revise our estimate of *P*_ϕ,*j*_ according to Equation (7). We then repeat this procedure iteratively (Figure S1). A convergence analysis shows that 2 iterations are sufficient to yield predictions on ϕ_*r*_ that varied by <0.1% (Figure S3). This fully characterises the relationship between ocular compliance and intraocular pressure, as determined using the Discrete Volume Method.

#### Calculation of Ocular Compliance Using the Step Response Method

Using the Step Response Method, ocular compliance is calculated by fitting Equation (8) to the time-varying measured pressure in response to a step change in applied pressure. As λ in Equation (8) is a function of γ (Equation 10), an iterative scheme that uses sequential estimates of τ_*j*_ to update λ_*j*_ is required, which we describe below. Note that λ_*j*_ should not be defined as a free parameter, as it is a function of numerous other system parameters (Equation 10).

To calculate ocular compliance at the *j*^*th*^ step, ϕ_*j*_, several additional parameters are required (β, *C*_*q*_, and γ). β was determined by fitting $Q\;=\;C_{r}\;{\left(P/P_{r}\right)}^{\beta }\;P$ to the *Q*_*j*_ and *P*_*j*_ data using non-linear regression as described previously (Sherwood et al., 2016). To estimate *C*_*q*_, we use the fact that *C*_*q*_ = *Q*_*j*_/(*P*_*a,j*_ − *P*_*j*_). Using linear regression, we calculate the slope of the *P*_*a,j*_ − *P*_*j*_ against *Q*_*j*_ relationship, allowing the intercept to be a small error term, *e*_*P*_*a*__:

(14) $$\begin{array}{l} P_{a,j}-P_{j}=\frac{Q_{j}}{C_{q}}-e_{P_{a}} \end{array}$$

For the present study, the term *e*_*P*_*a*__ was very small [< 0.05 mmHg, which is within the resolution of the pressure sensor (Sherwood et al., 2016)].

To determine γ and to account for the dependence of λ_*j*_ and τ_*j*_ upon ϕ_*j*_ (Equations 9 and 10), we used the following iterative scheme. We first calculated an initial estimate of τ_*j*_ by omitting terms of order (Δ*P*_*j*_/*P*_*j*_) and higher which is equivalent to setting λ_*j*_ = 0. Equation (9) gave a first estimate of ϕ_*j*_ from the initial estimate of τ_*j*_. We then fit Equation (3) to the estimated values of ϕ_*j*_ and *P*_*j*_, yielding an initial estimate of γ, which was used to update the value of λ_*j*_ based on Equation (10). The updated value of λ_*j*_ was used to repeat the fit of Equation (8), yielding updated values of τ_*j*_ and ϕ_*j*_ and, by Equation (3), updated values of γ and ϕ_*r*_. The process was repeated 5 times, which yielded an estimate of ϕ_*r*_ that varied by <0.1% (Figures S1, S3). When performing the fit with Equation (8), we defined *t* = 0 as the start of the step (i.e., when the actuator began moving), but excluded data from the fit during the time window when the actuator was moving. As the actuator speed was 3 mm/s, this time window lasted 5–10 s, depending on step size.

### *In vitro* Tests Using a Model Eye

In order to test the above methods, we first applied the analysis techniques to an *in vitro* model eye that approximates the outflow resistance and ocular compliance of a mouse eye. The model eye consists of a 100 mm length of glass capillary (CM Scientific) with an inner diameter of 50 ± 5 μ*m* and a length *L* of compliant vinyl tubing with a compliance ϕ_*L*_ and an inner diameter of 6.35 *mm* (Swagelok, UK). Tubing lengths of ~10, 30, 50, 70, 110, 150 and 230 *mm* were investigated. We first measured the system compliance, ϕ_*s*_, in the absence of the compliant tubing. Then after adding the compliant tubing and outflow capillary, we performed five to seven pressure steps of 3 *mmHg* to determine total compliance ϕ_*t*_ = ϕ*s* + ϕ_*L*_, where ϕ_*L*_ is the compliance of the tube (expected to be proportional to tube length). Average values of ϕ_*L*_ were then given by $\bar{\phi_{L}}=\bar{\phi_{t}}-\bar{\phi_{s}}$, with standard deviation $s_{L}=\sqrt{{s_{t}}^{2}+{s_{s}}^{2}}$. $\bar{\phi_{L}}$ was measured using both the Discrete Volume and Step Response Method, as described below.

*In vitro* experiments were carried out using an earlier version of the *iPerfusion* system that had a flow sensor with a larger hydraulic resistance (10 *mmHg*/(μ*l*/min); SLG64-0075, Sensirion) compared to that used for the *ex vivo* experiments (0.6 *mmHg*/(μ*l*/min); SLG150, Sensirion). The higher resistance flow sensor obviated the need for the upstream capillary added to slow the system response. Hence, for these experiments, *C*_*q*_ represented the hydraulic conductivity of the flow sensor alone.

#### *In vitro* Tests of a Model Eye Using the Discrete Volume Method

**Figure 2:**
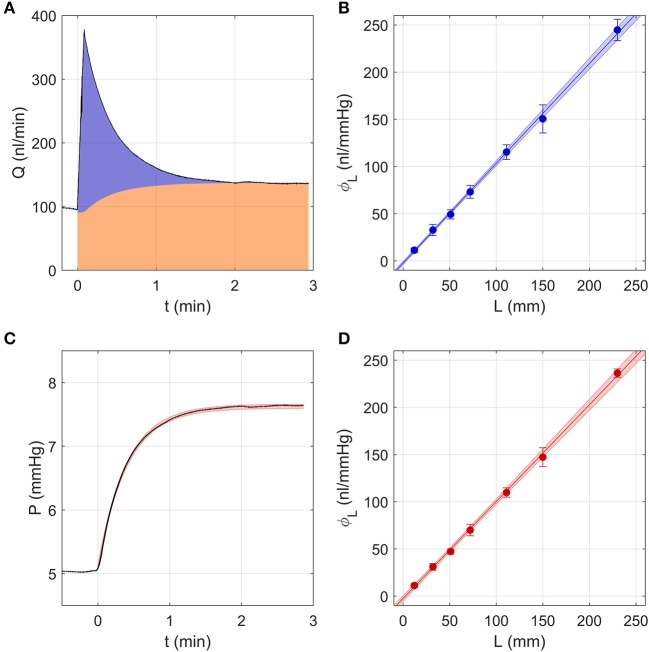
Testing the two analytical methods used to calculate ocular compliance using an *in vitro* model eye, consisting of a glass capillary and compliant tubing to represent aqueous humour outflow resistance and ocular compliance, respectively. **(A)** The Discrete Volume Method, showing a sample measured flow rate step response (black tracing) for compliant tubing of length *L* = 30 mm as pressure was changed from 5 to 8 mmHg. Area shaded in orange represents the fluid volume passing through the outflow capillary, while the blue area represents the volume filling the system and tubing compliances (see text). **(B)** Measured values of tubing compliance, ϕ_*L*_, vs. tubing length, *L*, determined by the Discrete Volume Method and fit by linear regression (Equation 15). **(C)** Step Response Method, showing a sample pressure tracing (black) for the step shown in panel a. Red shading indicates the 95% confidence intervals on the fit to the pressure response (Equations 8–9 with λ = 0). **(D)** Measured values of tubing compliance, ϕ_*L*_, vs. tubing length, *L*, determined by the Step Response Method, fit by linear regression (Equation 15). In **(B,D)**, the shaded regions represent the 95% confidence bounds on the fit and the error bars represent 2 SD.

Figure 2A shows a sample flow rate tracing over a single pressure step using the Discrete Volume Method. The area shaded in orange represents the volume that passed through the outflow capillary, equivalent to $\int_{0}^{T}C\;P\;dt$, where *C* represents the hydraulic conductance of the outflow capillary, measured as described in section Calculation of Ocular Compliance using the Discrete Volume Method. The area shaded in blue represents the volume that filled the combined compliance of the system and tubing, equivalent to $\int_{0}^{T}\left(Q-C\;P\right)\;dt$. Assuming a constant tubing compliance per unit length, *dϕ*_*L*_/*dL*, the measured values of $\bar{\phi_{L}}$ were analysed by weighted linear regression, with weights equal to the inverse variance, according to

(15) $$\begin{array}{l} \phi_{L}=\phi_{0}+\frac{d\phi_{L}}{dL}L \end{array}$$

where ϕ_0_ represents a systematic offset that would ideally equal zero. The fit yielded ϕ_0_ = −2.3 [−3.1, −1.4] *nl*/*mmHg* (mean [95% CI]) and *dϕ*_*L*_/*dL* = 1.06 [1.04, 1.07] *nl*/*mmHg*/*mm*, as shown in Figure 2B.

#### *In vitro* Tests of a Model Eye Using the Step Response Method

As the hydraulic conductance and compliance of the glass capillary and tubing should not change with pressure, Equations (8–10) were reduced to their zeroth order form, which is equivalent to setting λ_*j*_ and β to 0. Similarly, the value of *C* was calculated by linear regression of *Q*_*j*_ vs. *P*_*j*_, rather than by using Equation (4). These simplifications eliminated the need for the iterative procedure described in section Calculation of Ocular Compliance using the Step Response Method. The resulting fits obtained from the Step Response Method closely matched the pressure tracings measured using the model eye (Figure 2C). Averaging the predicted tubing compliances over all pressure steps and fitting $\bar{\phi_{L}}$ vs. *L* using Equation (15) yielded ϕ_0_ = −2.4 [−3.9, −1.0] *nl*/*mmHg* and *dϕ*_*L*_/*dL* = 1.03 [1.01, 1.04] *nl*/*mmHg*/*mm*, as shown in Figure 2D.

#### Comparing Results From the Discrete Volume and Step Response Methods Using a Model Eye

The Discrete Volume and Step Response Methods yielded values of ϕ_0_ and *dϕ*_*L*_/*dL* that were statistically indistinguishable. Further, both methods yielded estimates of *dϕ*_*L*_/*dL* with very small confidence intervals. This confirms that tubing compliance is a linear function of length, as assumed, and demonstrates that each method exhibits a high degree of repeatability, as indicated by the 95% confidence intervals being a small fraction (<7%) of the mean. For both cases, the estimated value of ϕ_0_ was small, but statistically different from zero, indicating that the measurement resolution is on the order of a few *nl*/*mmHg*. From this analysis, we conclude that the Discrete Volume and Step Response Methods are similarly valid and precise, at least for the simplified case of a model eye having constant compliance and outflow resistance.

### *Ex vivo* Experiments

#### Experimental Design

We conducted all measurements on paired contralateral mouse eyes. Our rationale was that we expected two eyes of a pair to exhibit similar values of ocular compliance, as previously observed for outflow facility (Sherwood et al., 2016). We examined 12 pairs of enucleated mouse eyes and measured the apparent variability in ocular compliance between contralateral eyes. We also compared the ocular compliance determined using the Discrete Volume and Step Response Methods and examined whether either method yielded a more precise measurement (i.e., tighter confidence intervals) of compliance. We then asked whether our methods could detect an imposed change in ocular compliance. For this purpose, we incubated one eye of 6 pairs with genipin, an agent known to crosslink collagen and increase corneoscleral stiffness (Campbell et al., 2017), while the contralateral eye was incubated in vehicle without genipin. We compared the difference between genipin-treated and vehicle-treated eyes against the measured variability between untreated contralateral eyes.

#### Animal Husbandry

All procedures were approved by the Institutional Animal Care and Use Committee at the Georgia Institute of Technology. C57BL/6J mice were purchased at 10 weeks of age from Jackson Laboratory and housed for a minimum of 7 days with a 12 h light/dark cycle, before euthanasia by CO_2_. Upon sacrifice, eyes were gently proptosed with forceps, and enucleated using curved scissors. The enucleated eyes were stored in PBS at room temperature for up to 30 min before cannulation.

#### Genipin Stiffening

After enucleation, treated and control eyes were, respectively, incubated in 15 mM genipin (078-03021; Wako Pure Chemical Industries Ltd, Richmond, VA) in PBS, or in PBS alone, for 30 min at 37°C. Eyes were cannulated and perfused immediately following the incubation period.

### Statistical Analysis

Our previous studies showed that outflow facility is log-normally distributed in mice (Sherwood et al., 2016). However, a Shapiro-Wilk test applied to the non-transformed or log-transformed values was unable to clearly establish whether ocular compliance was better described by a normal or log-normal distribution (*p* > 0.05). Nevertheless, log-normal distributions are common whenever a parameter must take on a positive value and depends multiplicatively on several other independently distributed parameters (Limpert et al., 2001). Based on this we treated ocular compliance as log-normally distributed, similar to outflow facility. All statistical analyses requiring normally distributed variables (mean, regression, *t*-test, correlation) were therefore calculated on log-transformed values. Comparison of paired data were evaluated using a 2-tailed paired *t*-test on log-transformed values of ocular compliance. Regression parameters and statistically averaged values are presented in terms of the mean and 95% confidence interval.

## Results

### Demonstration of the Two Methods to Measure Ocular Compliance

We first examine a representative sample analysed using both the Discrete Volume and Step Response Methods. The sample was chosen from the 12 pairs of untreated contralateral eyes and exhibited typical values for both ocular compliance and outflow facility.

#### A Representative Sample Analysed Using the Discrete Volume Method

**Figure 3:**
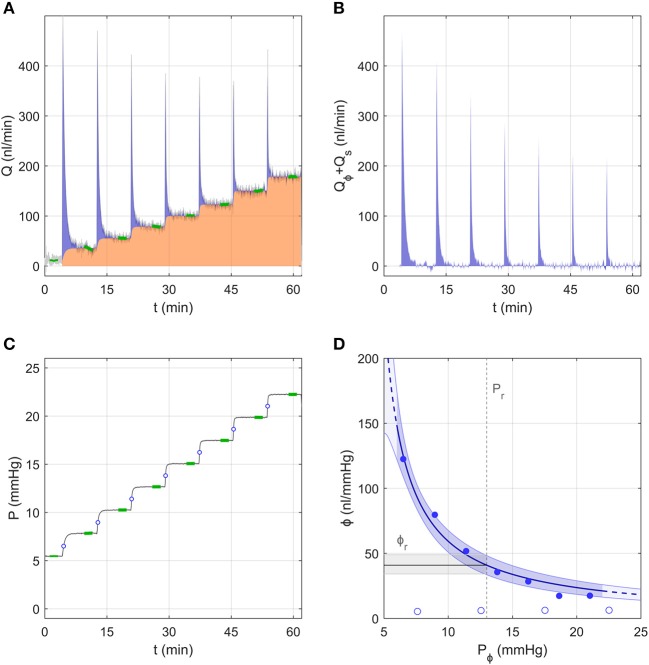
A representative data sample to demonstrate the analysis using the Discrete Volume Method. **(A)** The measured flow rate *Q* over 7 increasing pressure steps in an untreated mouse eye. Green highlights indicate the stable flow rate for each step. The area shaded in orange represents the volume passing through the outflow pathway, while the blue shaded area represents the “filling volume” that goes into expanding the system and tubing compliances (see text). **(B)** The flow rate tracing from panel a corrected to show only the component that enters the combined compliance of the system and eye, defined as *Q*_ϕ_ + *Q*_*s*_ = *Q* − *Q*_*r*_ (see Figure 1B). **(C)** The measured pressure tracing for the same experiment shown in panel a, with green highlights indicating the stable pressure for each step. Open circles indicate the value of *P*_ϕ,*j*_ at each step. **(D)** Ocular compliance, ϕ, plotted against pressure (filled circles). Blue curve represents the best fit by Equation (3), along with 95% confidence bounds (in blue shading). Black line and grey shading represent the estimated reference compliance, ϕ_*r*_, and its 95% confidence interval at a reference pressure, *P*_*r*_, of 13 mmHg (dashed vertical line). Open circles represent system compliance measured over a sequence of similar pressure steps prior to the ocular measurement.

Figure 3 shows the flow and pressure tracings for an individual eye analysed using the Discrete Volume Method, as pressure was incremented from ~5 to 23 mmHg over 7 steps. At the start of each pressure step, the flow rate increased immediately and decayed quickly, reaching a stable value (*Q*_*j*_) highlighted in green near the end of each step (Figure 3A). The area under the curve shaded in orange represents the volume that passed through the aqueous humour outflow pathway, defined as $\int_{0}^{T}C\;P\;dt$. The area shaded in blue represents the “filling volume” that went into expanding the combined compliances of the system and eye, defined as $\int_{0}^{T}\left(Q-C\;P\right)\;dt$. With each increasing pressure step, there was a decrease in the filling volume, indicating a decrease in ocular compliance with increasing pressure (because the system compliance remained constant; open circles, Figure 3D). To illustrate this point more clearly, Figure 3B shows only the component of the flow entering the filling volume, *Q* − *C P*.

Figure 3D shows the measured ocular compliance for each pressure step (filled symbols), plotted against the corresponding pressure *P*_ϕ,*j*_. Recall that, according to Equation (7), *P*_ϕ,*j*_ lies at an intermediate value between the starting and ending pressures for each step (indicated by open circles in Figure 3C). Consistent with the decrease in filling volume, there is a clear pressure-dependent decrease in ocular compliance. Also shown in Figure 3D is the fit of Equation (3), which for this representative sample yielded ϕ_*r*_ = 41 [34, 49] *nl*/*mmHg* and γ = −3.3 [−4.4, −2.3] *mmHg*, with *P*_*r*_ defined as 13 mmHg, chosen to match the population average IOP for C57BL/6J mice (Savinova et al., 2001). The fit is reasonably good, with the 95% confidence interval on ϕ_*r*_ covering a range of 36% of the estimated value (indicated by the grey band in Figure 3D).

#### A Representative Sample Analysed Using the Step Response Method

**Figure 4:**
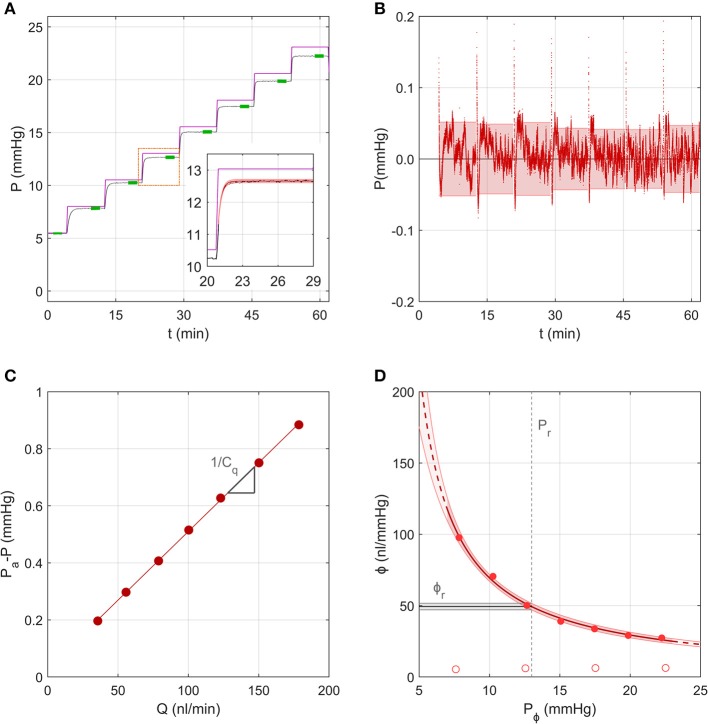
A representative sample to demonstrate the analysis using the Step Response Method, using data from the same experiment shown in Figure 3. **(A)** The measured pressure *P* over 7 increasing pressure steps in an untreated mouse eye (black tracing; data repeated from Figure 3C). Purple line shows the applied pressure, *P*_*a*_. Green highlights indicate the stable pressure for each step. Inset shows pressure data from a single step (black tracing) fit by the analytical model (red shading) given by Equations (8)–(10). **(B)** Residuals between the model fit and pressure tracing over all steps, with 95% confidence intervals on the residual for each step indicated by the shading. **(C)** Pressure drop across the capillary and flow sensor plotted vs. the measured flow rate, with a linear fit according to Equation (14) yielding estimated values of the slope (1/*C*_*q*_, see section Calculation of Ocular Compliance using the Step Response Method for details). **(D)** Ocular compliance, ϕ, plotted against pressure, *P*_ϕ_ (filled circles). Red curve represents the best fit by Equation (3), along with 95% confidence bounds (in red shading). Black line and grey shading represent the estimated reference compliance, ϕ_*r*_, and its 95% confidence interval at a reference pressure, *P*_*r*_, of 13 mmHg (dashed vertical line). Open circles represent system compliance reproduced from Figure 3D.

Figure 4 shows the same representative sample from section Measurement of the System Compliance analysed using the Step Response Method. For each step, the pressure tracing matched closely the form of the analytical solution given by Equations (8–10) (Figure 4A inset). Residuals from the fit were typically <0.05 mmHg, indicating excellent fitting quality, but exhibited a repeatable time-dependent decrease over each step, which was most apparent at lower pressures (Figure 4B). This indicates that some subtle aspect of the ocular response was not fully captured by the analytical model, which may be partly attributable to the ramp period when the actuator is moving causing deviation from the ideal step response. Figure 4C shows the fit used to estimate the combined conductance of the capillary and flow sensor, yielding *C*_*q*_ = 208 *nl*/min/*mmHg* (see Equation 14).

Figure 4D shows the measured ocular compliance, ϕ_*j*_, that applies at the stable pressure *P*_*j*_ for each step, along with the corresponding fit by Equation (3), which yielded ϕ_*r*_ = 49[47, 52] *nl*/*mmHg* at *P*_*r*_ = 13 *mmHg* and γ = −2.7[−3.4, −2.0] *mmHg*. The predicted values of ϕ_*r*_ and γ were comparable to those obtained in section Measurement of the System Compliance), however the confidence interval on ϕ_*r*_ was smaller with the Step Response Method (10% of the estimated value of ϕ_*r*_ compared to 36% using the Discrete Volume Method).

### Comparison Between Methods Used to Measure Ocular Compliance

**Figure 5:**
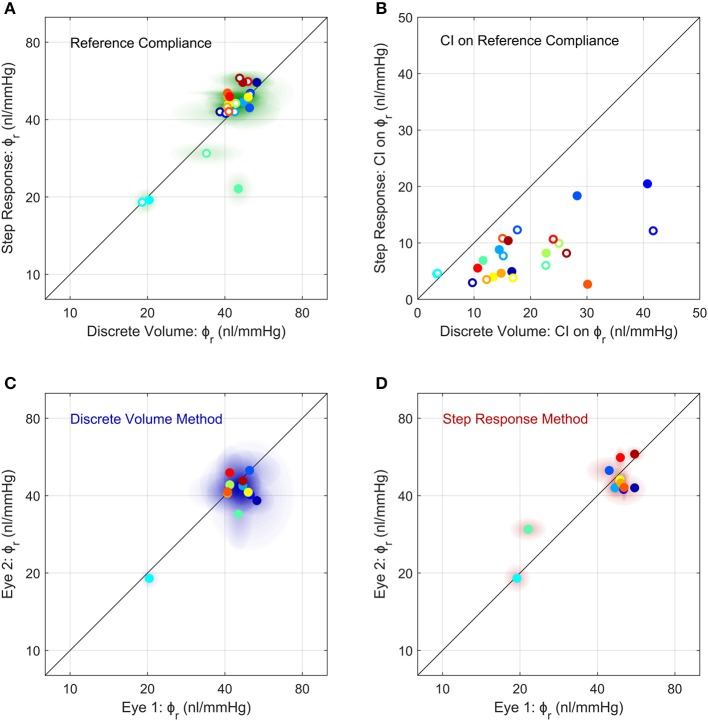
Comparison of the measurements of ocular compliance between the two methods **(A,B)** and between contralateral eyes **(C,D)**. **(A)** The reference ocular compliance, ϕ_*r*_, calculated by the Step Response Method plotted against that calculated by the Discrete Volume Method. Each data point represents an individual eye (*N* = 24) from 12 mice (separate colours), with filled vs. open circles identifying the two eyes from a given mouse. Shaded regions surrounding each data point represent the 95% confidence intervals on each estimate of ϕ_*r*_. Figure S4 shows a Bland-Altman plot of the same data. **(B)** The 95% confidence intervals on the estimated values of ϕ_*r*_ shown in panel a, using the same colour scheme as **(A)**. **(C,D)** A comparison of ϕ_*r*_ between contralateral eyes measured using the Discrete Volume Method **(C)** and Step Response Method **(D)**. Each data point represents an individual mouse, with ϕ_*r*_ from contralateral eyes plotted on each axis. Shading represents 95% confidence intervals. The colour scheme is the same as in **(A,B)**. The unity line, representing exact parity, is shown in black for each panel.

We then compared between measurements of ϕ_*r*_ obtained using the Discrete Volume and Step Response Methods (Figure 5A). Each method of analysis was performed on 24 untreated eyes from 12 mice, with each eye considered independently (i.e., ignoring any correlation between contralateral pairs). There was no difference in the population-averaged value of ϕ_*r*_ between the two methods (41 [37, 46] *nl*/*mmHg* vs. 42 [37, 49] *nl*/*mmHg*). However, the 95% confidence intervals on ϕ_*r*_, which are indicative of measurement precision, were lower for the Step Response Method, consistent with section Demonstration of the Two Methods to Measure Ocular Compliance (Figure 5B). The median confidence interval was 7 *nl*/*mmHg* for the Step Response Method and 16 *nl*/*mmHg* for the Discrete Volume Method. Figure S4 shows a Bland-Altman analysis of the same data, which demonstrates that no significant correlation between the two techniques was detected (*R*^2^ = 0.15, *p* = 0.06).

### Comparison of Ocular Compliance Between Contralateral Eyes

We then examined the correlation in ocular compliance between contralateral eyes, as measured by each method. For the Discrete Volume Method (Figure 5C), the Pearson's correlation coefficient for ϕ_*r*_ was *R*^2^ = 0.49 (*p* = 0.011) indicating a moderately significant correlation with approximately half of the variance attributable to variability between contralateral eyes. For the Step Response Method (Figure 5D), the correlation was significantly stronger, with *R*^2^ = 0.71 (*p* = 0.0006), indicating that only 30% of the total variance was attributable to variability between paired eyes. The statistically significant correlation observed using either method supports the use of paired experimental design when studying ocular compliance. However, the stronger correlation obtained with the Step Response Method suggests that this approach may provide more precise measurements of ocular compliance relative to the Discrete Volume Method.

### Effects of the Stiffening Agent Genipin on Ocular Compliance

**Figure 6:**
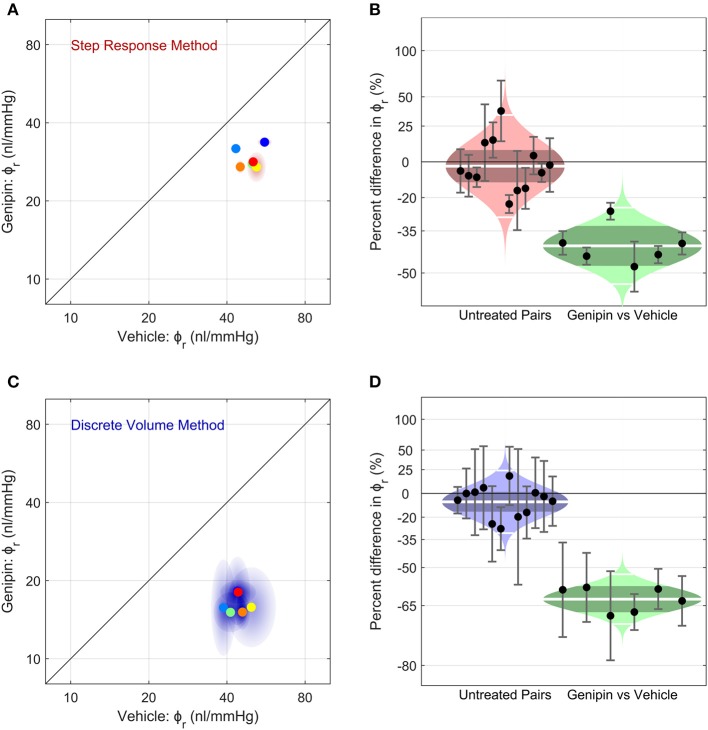
The effect of corneoscleral stiffening on ocular compliance using **(A,B)** the Step Response Method or **(C,D)** the Discrete Volume Method. **(A,C)** Scatter plots, showing the reference ocular compliance, ϕ_*r*_, measured in the genipin-treated eye plotted against that measured in the contralateral vehicle-treated eye, calculated by the Step Response Method **(A)** or the Discrete Volume Method **(C)**. Each data point represents an individual mouse (*N* = 6; separate colours) and shaded regions represent the 95% confidence interval on each estimated value of ϕ_*r*_. The unity line (black) represents exact parity between contralateral eyes. **(B,D)** Cello plots, showing the relative difference in ϕ_*r*_ between untreated contralateral eyes (left; *N* = 12 pairs) and between genipin-treated and vehicle-treated contralateral eyes (right; *N* = 6). For the untreated pairs (reproduced from Figure 5), there was no significant difference in ϕ_*r*_ between contralateral eyes using either the Step Response (*p* = 0.52) or Discrete Volume Method (*p* = 0.09). In contrast, for genipin treated eyes, there was a significant difference in ϕ_*r*_ relative to the vehicle-treated contralateral eye using either the Step Response (*p* = 0.0001) or Discrete Volume Method (*p* < 10^−5^). Shaded regions represent the best log-normal distribution that fits the data. The central white line represents the geometric mean, while the darker shading represents the 95% confidence interval on the geometric mean. The outer white lines on each distribution represent the limits encompassing 95% of the data. Data points represent the relative difference for an individual mouse, while the error bars represent the 95% confidence interval on that measurement.

Finally, we examined whether we were able to measure a change in ocular compliance following treatment with genipin, a collagen-crosslinking agent that should stiffen the corneoscleral shell and thereby decrease ϕ_*r*_. Figure 6A shows a scatterplot of ϕ_*r*_ values for genipin-treated vs. vehicle-treated contralateral eyes (*N* = 6 pairs), as measured using the Step Response Method. Genipin treatment decreased ϕ_*r*_ in every pair, evidenced by all data points falling below the unity line (which represents perfect agreement between contralateral eyes). On average, ϕ_*r*_ in the genipin-treated eye was lower (−41[−48, −33]%) compared to the contralateral vehicle-treated eye (*p* = 0.0001; Figure 6B right). In comparison, the difference in ϕ_*r*_ between untreated control eyes was −3 [−12, 8]% (*p* = 0.57; Figure 6B left). Analysis using the Discrete Volume Method yielded a similar relative decrease in ϕ_*r*_ between the genipin and vehicle-treated contralateral eyes (−63 [−67, −58]%; *p* < 10^−5^; Figures 6C,D). In contrast, there was no significant difference between untreated contralateral eyes (−8 [−16, 1]%; *p* = 0.09) analysed using the Discrete Volume Method. Measured values of ϕ_*r*_ and γ are provided in Table 1.

**Table 1:** Measured values of ϕ_*r*_ and γ from C57BL/6J eyes treated with either genipin or vehicle (mean [95% CI]; *N* = 6; *P*_*r*_ = 13 *mmHg*).

	**Discrete volume method**		**Step response method**	
	**ϕ_*r*_ [nl/mmHg]**	**γ [mmHg]**	**ϕ_*r*_ [nl/mmHg]**	**γ [mmHg]**
Vehicle-treated	43 [41, 46]	−2.85 [−2.91, −2.80]	49 [46, 52]	−2.44 [−2.50, −2.38]
Genipin-treated	16 [15, 17]	−3.11 [−3.17, −3.07]	29 [27, 31]	1.23 [1.17, 1.29]

## Discussion

Ocular compliance describes the pressure-volume response of the eye and is important in a number of physiological and pathological conditions, such as glaucoma. In this study, we developed methods to accurately measure ocular compliance in enucleated mouse eyes, a species widely-used in ophthalmic research. Using the *iPerfusion* system, the eye is exposed to small incremental steps in pressure, and the resulting flow rate and pressure response of the eye are analysed to calculate ocular compliance. Our analysis accounts for the strong dependence of ocular compliance on intraocular pressure. Our data demonstrate that ocular compliance is tightly correlated between contralateral eyes of individual mice. Moreover, our data demonstrate an expected decrease in ocular compliance following treatment with genipin, a crosslinking agent that increases corneoscleral stiffness. This work also demonstrates that the *iPerfusion* system, which was originally developed to measure outflow facility (Sherwood et al., 2016), can also be used to accurately measure pressure-dependent ocular compliance in mice. With relatively minor modifications, the approach could be generalised to *in vivo* measurements or to eyes from other species or other tissues.

### Accuracy and Precision of the Two Methods to Measure Ocular Compliance

We proposed two different methods to assess ocular compliance. The Discrete Volume Method relies on numerical integration of flow rate data to determine the filling volume that enters the eye during a step change in pressure. The Step Response Method, in contrast, relies on fitting an analytical solution to the measured pressure response following a step change in applied pressure. Both methods yielded nearly identical values of compliance when applied to an *in vitro* model system that included a defined length of compliant tubing, with a measurement uncertainty of a few *nl*/*mmHg*. Similarly, both methods yielded consistent estimates of the ocular compliance for C57BL/6 mice. The congruence in measured values obtained using two different methods in two distinct applications (*in vitro* and *ex vivo*) demonstrates complementarity and supports the claim that each approach can accurately measure ocular compliance.

The Step Response Method produced tighter estimates of ocular compliance with smaller confidence intervals relative to the Discrete Volume Method. The Step Response Method also yielded a stronger correlation between contralateral eyes of individual mice. This evidence suggests that the Step Response Method may provide greater precision relative to the Discrete Volume Method when measuring ocular compliance. However, the Step Response Method assumes that the form of the ocular *P* − *V* relationship is consistent with Equation (3). Any deviations in the true *P* − *V* relationship from that implied by Equation (3) would thereby introduce inaccuracies in the predicted value of ocular compliance. In contrast, the Discrete Volume Method does not rely entirely on a particular form of the *P* − *V* relationship, such that estimates of ocular compliance obtained by this method would be less sensitive to deviations from Equation (3). However, determination of the pressure *P*_ϕ,*j*_, which corresponds to the measured value of ocular compliance, indeed depends on the particular form of the *P* − *V* relationship (see Equation 7). As *P*_ϕ,*j*_ lies between the initial and final values of intraocular pressure during the step (*P*_*j*−1_ < *P*_ϕ,*j*_ < *P*_*j*_), any uncertainty in *P*_ϕ,*j*_ that arises due to an undefined *P* − *V* relationship may be minimised by using smaller pressure steps.

The greater precision of the Step Response Method is attributable to its reliance primarily on the pressure transient, while the Discrete Volume Method requires both pressure and flow transients. Immediately after the leading edge of the applied pressure step, the flow initially spikes, and then declines rapidly. Thus, the precision of the Discrete Volume Method that is based on flow is limited by the temporal resolution of the initial flow spike, which is often difficult to capture, particularly for lower values of ocular compliance. Failure to fully resolve the pressure spike would underestimate the filling volume and may explain why the Discrete Volume Method yielded smaller estimates of ϕ_*r*_ for the genipin-treated eyes relative to the Step Response Method (Table 1). In contrast, the pressure response changes more slowly, rising from an initial pressure without a spike, such that the pressure response may be more fully captured to yield more precise estimates of compliance by the Step Response Method. Alternatively, it may be possible to improve the precision of either method, but the Discrete Volume Method in particular, by using a higher resistance capillary or flow sensor to prolong the transient response, thereby enabling greater resolution of the flow or pressure response. This would come at the expense of longer perfusions but would have the added benefit of reducing the contribution of the ramp period during which time the pressure changes between steps.

### Comparison Against Previous Methods to Measure Ocular Compliance

Both the Discrete Volume and Step Response Methods analyse ocular compliance using the applied pressure approach, in which the eye is pressurised in incremental steps and the resulting changes in pressure and flow are measured. This differs from nearly all previous methods to measure ocular compliance or ocular rigidity, which primarily used the applied volume approach, in which defined volumes of fluid are injected into the eye and the resulting change in pressure is measured (Pallikaris et al., 2005; Lei et al., 2011; Stockslager et al., 2016; Ficarrotta et al., 2018) (analogous to an impulse response). Although the applied volume approach is valid in principle, practical application requires isolating the response of the eye from that of the measurement system and accounting for the pressure-dependence of ocular compliance itself. As described by Campbell et al. (2018) the pressure response following a bolus injection depends critically on the dynamics of the external fluidic system that is coupled to the eye for the purpose of measuring ocular compliance. Any fluidic system will have its own intrinsic hydraulic resistances and compliances and will thereby alter the measured response to a step change in applied flow or pressure. Isolating the behaviour of the eye from that of the system, and hence accurately measuring ocular compliance, requires an accurate model of the system dynamics (e.g., an equivalent circuit diagram as shown in Figure 1B) and knowledge of individual system resistances and compliances. The dynamics of the fluidic measurement system have not typically been accounted for in prior studies of ocular compliance, particularly in smaller mammals (e.g., Lei et al., 2011; Ficarrotta et al., 2018).

Furthermore, with the notable exception of Campbell et al. (2018), few studies have accounted for the considerable pressure-dependence of ocular compliance. As ϕ can decrease 3-fold or more between 8 and 20 mmHg, not accounting for this pressure-dependence can lead to significant inaccuracies in the assessment of ocular compliance. This complication is particularly important when using the applied volume approach with smaller eyes (e.g., mice, rats, or tree shrews) when a relatively small injected volume could lead to significant pressure spike, at which point ϕ may change several-fold in a very short period of time. Resolving ϕ and its corresponding pressure *P*_ϕ_ [see Campbell et al. (2018) for details] becomes more difficult as the magnitude of the pressure spike increases, although this effect could be minimised by reducing the injected volume. Similarly, values of ϕ should always be reported with their corresponding value of *P*_ϕ_.

Some authors (Li et al., 2016; Stockslager et al., 2016) have proposed methods to measure ocular compliance and/or outflow facility based on the decay of intraocular pressure following a bolus volume injection. Typically, these methods use exponential fits, wherein a single exponential time constant is extracted and related to outflow facility and ocular compliance, which are both assumed to remain constant. However, as both outflow facility and ocular compliance change significantly with pressure, the accuracy of the pressure-decay technique to measure parameters of ocular biomechanics is questionable.

### Relationship Between Ocular Compliance and Corneoscleral Elastic Modulus

Ocular compliance is an extensive property that depends on the elastic properties of the corneoscleral shell as well as the dimensions of the eye. Thus, when comparing ocular compliance between individuals or groups, one must account for differences in eye volume and/or corneosceral thickness. Elastic modulus, in contrast, is an intensive property that is independent of morphology and describes the aggregate material properties of the corneoscleral shell itself. It is thus useful to relate ocular compliance to corneoscleral elastic modulus, which can be compared directly between different groups or species.

Purslow and Karwatowski (1996) described the *P* − *V* relationship of a thin-walled corneoscleral shell in terms of its radius *R*, thickness *h* and “incremental” Young's modulus *E*_*p*_ according to

(16) $$\begin{array}{l} \frac{dP}{dV}=\frac{1}{4\;\pi \;R^{3}}\left(\frac{4\;E_{p}\;h}{R}-3P\right) \end{array}$$

This relationship assumes that the corneoscleral shell is perfectly spherical, purely elastic (with a pressure-dependent elastic modulus), isotropic, incompressible, thin-walled and homogeneous in regard to elastic modulus and thickness. The validity of these assumptions is questionable. For example, the cornea has a higher curvature than the sclera, the sclera is thinner near the equator than at the posterior pole, and the corneoscleral shell exhibits anisotropic and heterogeneous elastic properties. However, the relationship allows one to determine the Young's modulus of a purely elastic, homogeneous and isotropic spherical shell that would exhibit the same value of ocular compliance as an eye of given dimensions at a given pressure. This provides a useful benchmark to compare the “effective” corneoscleral elastic modulus between groups of eyes of that may have different dimensions.

**Figure 7:**
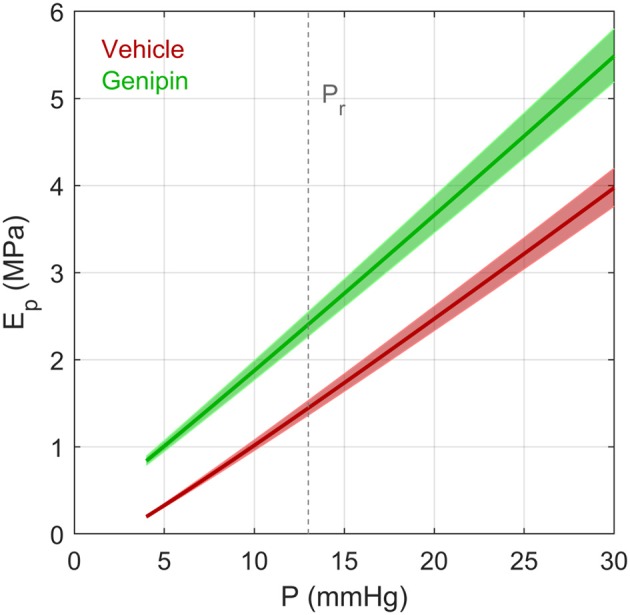
The effective Young's modulus *E*_*p*_ of an elastic spherical shell having the same compliance as that of a typical vehicle-treated and genipin-treated eye from a young adult C57BL/6 mouse calculated using the Step Response Method. *E*_*p*_ was determined based on Equations (17) and (3), assuming a radius *R* = 1.6 *mm* and thickness *h* = 0.04 *mm* (defined at 13 mmHg). Note that *R* and *h* change as a function of pressure due to the changing volume of the eye and constraints of incompressibility applied to the corneoscleral shell. Values of ϕ_*r*_ and γ were defined based on values reported in Table 1. Shaded regions represent uncertainty due to 95% confidence intervals on ϕ_*r*_. Dashed vertical line represents the reference pressure, *P*_*r*_, defined to be 13 mmHg.

Recognising that *dP*/*dV* = 1/ϕ allows Equation (16) to be rearranged to give an expression for *E*_*p*_

(17) $$\begin{array}{l} E_{p}=\frac{3\;R}{4\;h}\left(\frac{V}{\phi }+P\right) \end{array}$$

where *V* = 4π*R*^3^/3 is the volume of the corneoscleral shell. Note that since *V*/ϕ ≫ *P* for mouse (as well as for human) eyes, *E*_*p*_ can be approximated as (π *R*^4^)/(ϕ *h*). As ϕ decreases with increasing pressure, *E*_*p*_ is not constant but increases with pressure. Using the form of ocular compliance given by Equation (3), empirical values of ϕ_*r*_ and γ obtained from our data (Table 1), and previously reported data on thickness and radius [*h* = 0.04*mm* and *R* = 1.6 *mm* (Myers et al., 2010)] we can determine an effective pressure-dependent Young's modulus for vehicle- and genipin-treated eyes (Figure 7). This analysis shows that genipin crosslinking resulted in ~65% increase in the effective corneoscleral elastic modulus at 13 mmHg. We point out that the form of the ocular compliance given by Equation (3) is consistent with a linear pressure-dependent increase in the effective Young's modulus of the corneoscleral shell.

### Limitations and Future Work

The relationship describing how ocular compliance changes with pressure (Equation 3) is based on the form of the pressure-volume relationship reported by Ethier et al. (2004), which is consistent with the common Friedenwald relationship when γ = 0. In our analysis, γ was treated as a free parameter when fitting Equation (3) to the compliance-pressure data. With the exception of the genipin-treated eyes using the Step Response Method (Table 1), the fitted values of γ were always negative. However, in the derivation of Equation (3) (see Supplementary Material S1), γ is defined as 2*Ah*/*R*, where *h* is the corneoscleral thickness, *R* is the eye radius, and *A* is proportional to the corneoscleral elastic modulus. As these parameters should all be positive, negative values of γ are unexpected and not physically meaningful. Note also that Equation (3) yields a compliance that tends to infinity as *P* decreases toward −γ and becomes negative for *P* < −γ. Hence, as the fitted values of γ are largely negative, there are positive values of *P* (typically <3 mmHg per Table 1) for which Equation (3) may not apply. The exact reason for the negative estimates of γ is presently unclear but could be related to assumptions used in the derivation (e.g., small strain approximation, isotropy, heterogeneity) and/or limitations in the constitutive model for collagen [e.g., neglecting crimp (Grytz et al., 2014; Jan et al., 2018)]. Regardless, despite the difficulty of interpreting the empirical value of γ, the quality of the resulting fits using Equation (3) is excellent and therefore appropriate for capturing the pressure dependence of ϕ and for comparing ϕ_*r*_ between different groups. Future work should aim to resolve any ambiguity surrounding the mathematical form of the pressure-volume relationship and describe how γ is related to the biomechanical properties of the corneoscleral shell.

By definition, ocular compliance measures the combined pressure-volume behaviour of the entire eye. When applied to *ex vivo* eyes, this response is largely attributable to expansion of the corneoscleral shell. As the cornea and sclera may have different mechanical properties, it is not possible to infer properties of either the cornea or sclera based on ocular compliance measurements alone. However, in combination with imaging techniques, such as digital image correlation (Myers et al., 2010; Campbell et al., 2017), it may be possible to use strain mapping to infer properties of the cornea or sclera (or both). Furthermore, fluid movement within the choroid may influence the pressure-volume response. The choroidal response is expected to be negligible *ex vivo* because blood pressure is eliminated, but changes in choroidal blood volume may be significant if the technique were applied *in vivo*.

The current model does not include viscoelastic behaviour of the corneoscleral shell, which would alter the dynamic response of the eye. In order to explore the viscoelastic properties of the corneoscleral shell, a frequency domain approach could be used, where an oscillatory pressure or flow is applied to the eye, and the resulting phase lag and amplitude of the pressure or flow response could be used to infer viscoelastic storage and loss moduli.

Due to the pressure-dependence of ocular compliance, numerical iterations were necessary to calculate ϕ_*r*_. For the present set of data, an average change of <0.1% between subsequent iterations was achieved for both the Discrete Volume Method (by 2 iterations) and the Step Response Methods (by 5 iterations). Note however, that these values are highly dependent on system dynamics, and hence we advise that numerical convergence be evaluated for each new application of the technique.

In principle, the techniques described here could be translated to eyes of other species. However, ocular compliance increases with size, which would prolong the time response. To reduce the response time, it may therefore be necessary to remove the capillary upstream of the flow sensor and to use a flow sensor with a smaller resistance. Using the circuit model shown in Figure 1B, it is possible to analyse the system dynamics so as to optimise the response time for a particular set of parameter values.

## Conclusions

We have developed two methods to measure ocular compliance in mice using *iPerfusion*. Ocular compliance decreases sharply with pressure, yet the reference ocular compliance at physiological IOP is tightly correlated between contralateral eyes. Ocular compliance decreases significantly in response to collagen crosslinking by genipin. The methodology reported here provides highly accurate and precise measurements of ocular compliance that are useful for ocular phenotyping, thereby improving our understanding of ocular biomechanics and ocular physiology.

## Data Availability Statement

The datasets generated for this study are available on request to the corresponding author.

## Ethics Statement

All procedures were approved by the Institutional Animal Care and Use Committee at the Georgia Institute of Technology. The protocol number was A17023.

## Author Contributions

JMS, EMB, AJF, CRE, and DRO contributed to conception and design of the study. EMB carried out *ex vivo* experiments. JMS carried out *in vitro* experiments, developed the ocular compliance measurement and processing methodology, and performed the statistical analysis. JMS, DRO, CRE, and KP carried out the mathematical formulation. JMS and EMB wrote the first draft of the manuscript. CRE and DRO wrote sections of the manuscript. All authors contributed to manuscript revision, read and approved the submitted version.

### Conflict of Interest

JMS receives consultancy income for building and providing iPerfusion systems. The remaining authors declare that the research was conducted in the absence of any commercial or financial relationships that could be construed as a potential conflict of interest.

## Footnotes

^1^Note that *P*_*a*_ changes negligibly during a single step within a perfusion, as the cross-sectional areas of the reservoir and eye bath are sufficiently large relative to the small volumes of fluid perfused.
