# Supplementary material for: Measurement of Ocular Compliance Using iPerfusion
Source: Front Bioeng Biotechnol. 2019 Oct 25;7:276. doi: 10.3389/fbioe.2019.00276 (PMC6823226; doi:10.3389/fbioe.2019.00276)
Supplement: Supplementary file 1 [file Image_1.pdf]

frontiers

Supplementary Figures

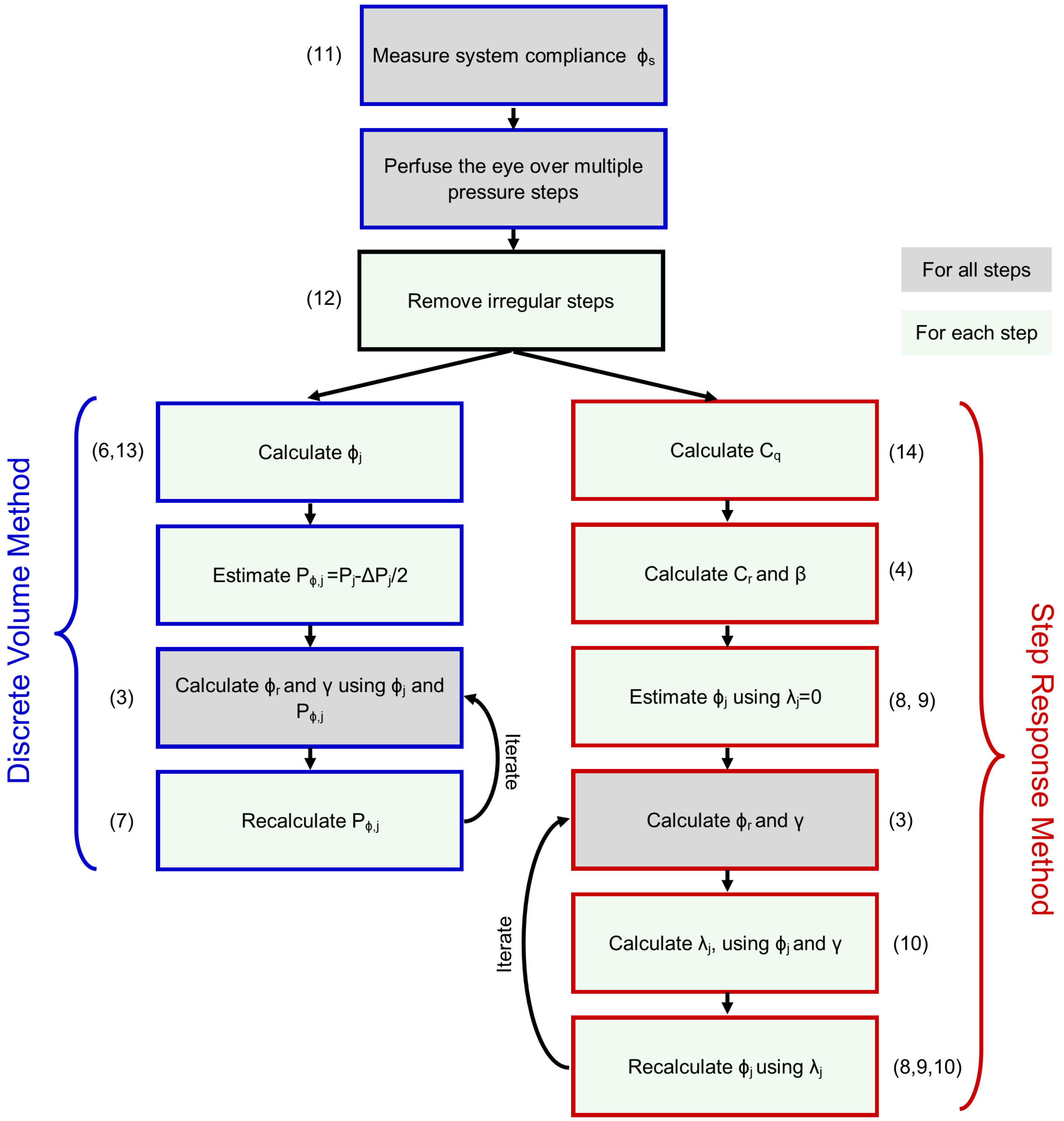

Figure S1. Block diagram summarising the procedure to calculate ocular compliance using the Discrete Volume and Step Response Methods. Numbers in brackets represent the relevant equations in the main text.

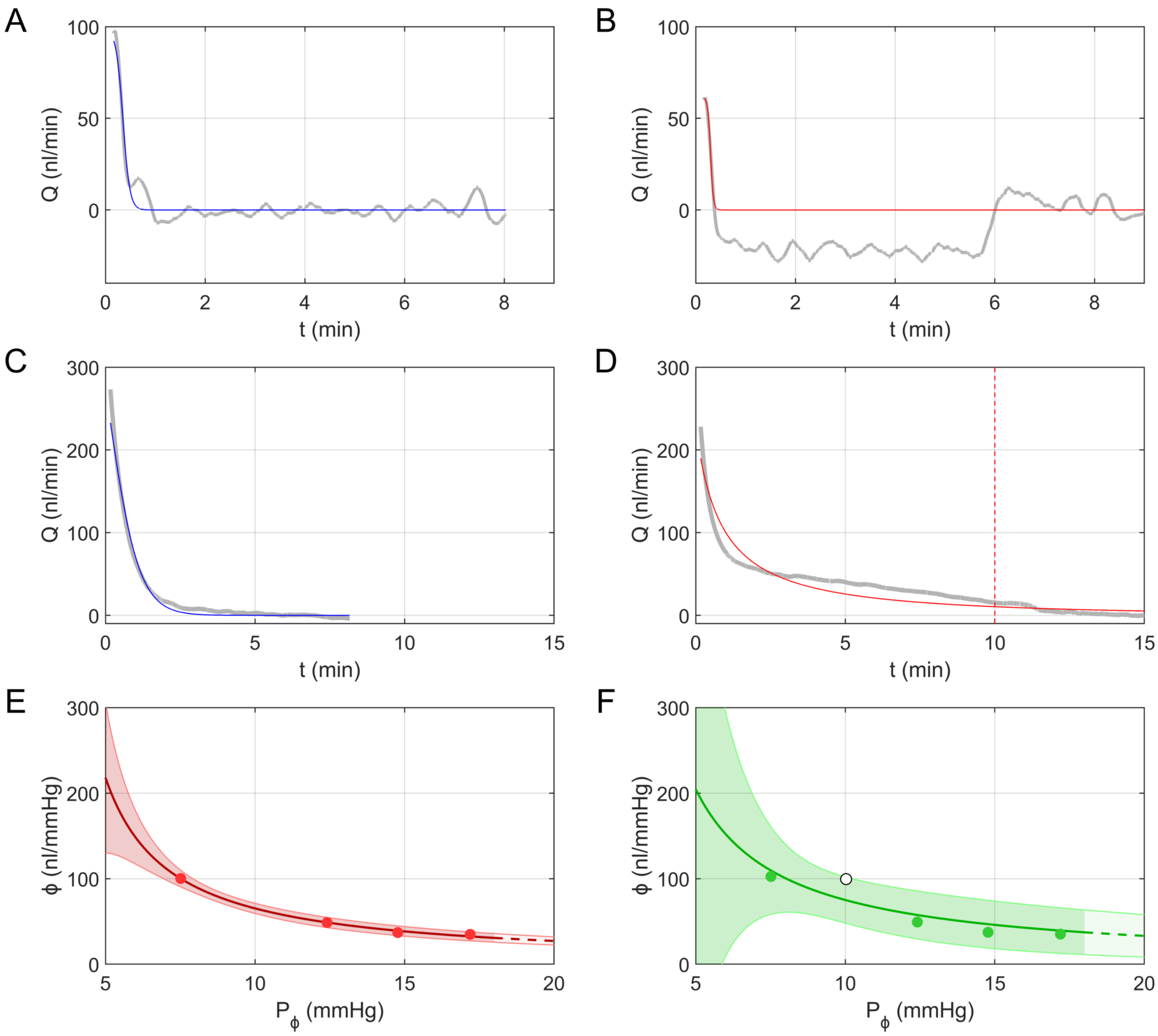

Figure S2. Examples of regular and errant step responses, where errant responses were removed from further analysis. Grey lines show raw flow rate data, and smooth coloured lines show fits to Equation 12 multiplied by $Q_{max} - Q_j$ to re-dimensionalise. Panels A and B show flow rate versus time traces following a pressure step in a genipin-treated eye. The step response shown in panel B exhibits a rapid jump in flow rate at approximately 6 minutes and was removed from the analysis due to exceeding the residual threshold, while the step response in panel A does not exhibit a jump and was included. Panels C and D show flow rate versus time traces following a pressure step in a vehicle-treated eye. The step response shown in panel D takes 15 minutes to reach stability (longer than the 10 minute inclusion criteria, represented by the dashed vertical line), and was thus removed, while the step response in panel C was included in the analysis. Panels E and F demonstrate the effect of errant steps on fitting the compliance-pressure relationship. The errant step (empty marker in panel F) is the case shown in panel D. Excluding the errant step produces a good fit (panel E), while including the errant step yields a poor fit (panel F). The coloured regions indicate the 95% confidence bounds on the fits to Equation 3.

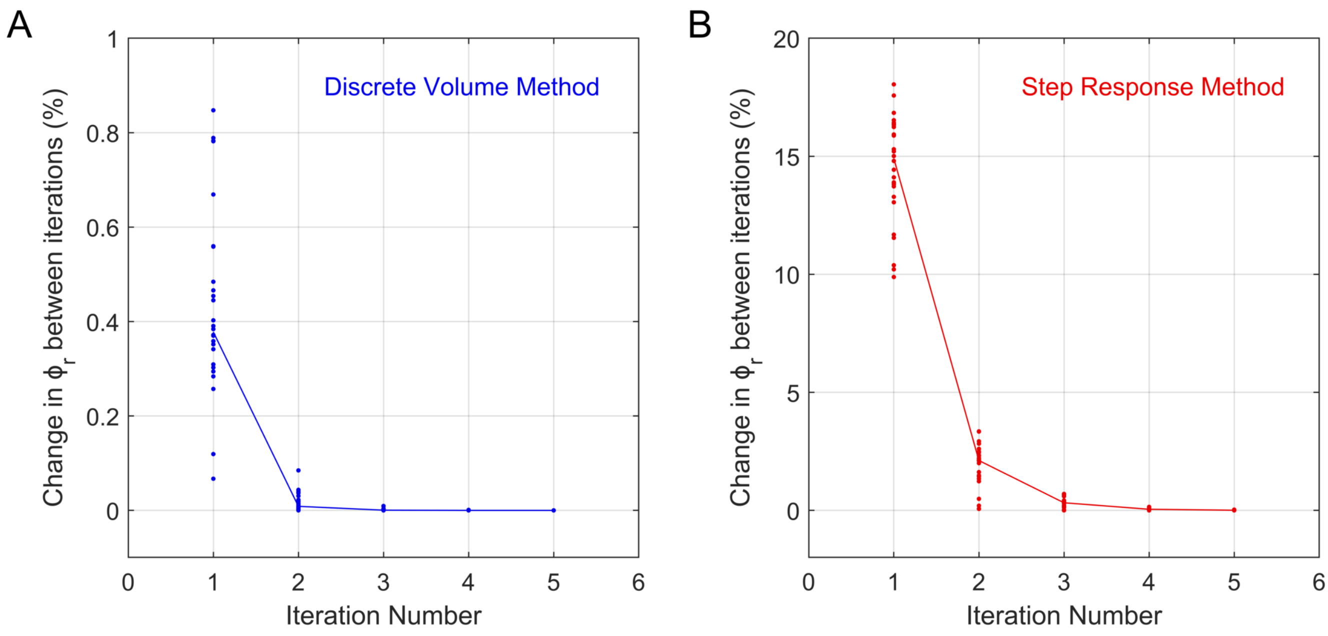

Figure S3. Analysis of convergence of the iteration procedures. A Discrete Volume Method. B Step Response Method. Data points represent the relative change in ϕ_r for individual eyes over subsequent iterations, whilst lines show the trend in the mean value as a function of iteration number.

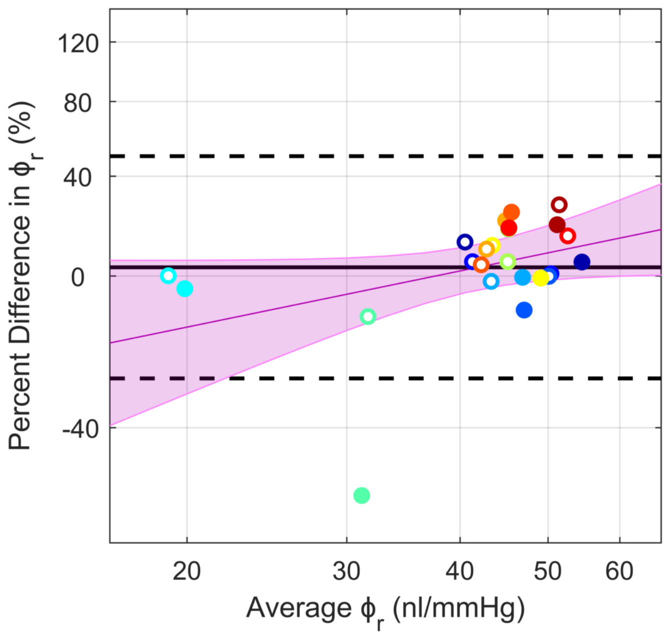

Figure S4. Bland-Altman analysis for the reference ocular compliance, ϕ_r, measured by the Discrete Volume and Step Response Methods applied to the same data for individual untreated eyes shown in Figure 5 of the main text (N = 24). The average percent difference was 1%, with two standard deviations covering the range -30 to +47%. No correlation was detected between ϕ_r measured by the two techniques ($R^2 = 0.14, p = 0.07$), as visualised by the purple line and shading representing the best linear fit and the 95% confidence bounds. Filled and open circles of the same colour represent contralateral eyes from an individual mouse.
