# Supplementary material for: Measurement of Ocular Compliance Using iPerfusion
Source: Front Bioeng Biotechnol. 2019 Oct 25;7:276. doi: 10.3389/fbioe.2019.00276 (PMC6823226; doi:10.3389/fbioe.2019.00276)
Supplement: Supplementary file 2 [file Data_Sheet_1.pdf]

Supplementary Material

S1 COMPLIANCE-PRESSURE RELATIONSHIP OF THE EYE

According to Ethier et al. (2004), the pressure-volume relationship of the eye can be written as

$$\ln\left(\frac{P+\gamma}{P_{r,\phi}+\gamma}\right) = \frac{\alpha}{3V_{r,\phi}}\left(V - V_{r,\phi}\right) \tag{S1}$$

where $\gamma = 2Ah/R$, and $P_{r,\phi}$ and $V_{r,\phi}$ represent the values of P and V at a specific reference state. R and h represent the radius and thickness of the corneoscleral shell. A and α represent the material properties of the corneoscleral shell, defined based on the Fung constitutive relation for collagenous tissues describing stress σ in terms of strain ε according to $\sigma = A\left(e^{\alpha\varepsilon} - 1\right)$.

Expressing Equation 1 in terms of V and differentiating with respect to P yields an expression for the ocular compliance given by

$$\phi = \frac{3V_{r,\phi}}{\alpha}\frac{1}{P+\gamma} \tag{S2}$$

If we define a reference ocular compliance, ϕ_r that applies at $P = P_{r,\phi}$, then ϕ can be written in terms of ϕ_r as

$$\phi = \phi_r\left(\frac{P_{r,\phi}+\gamma}{P+\gamma}\right) \tag{S3}$$

This expression describes the ocular compliance relationship as a function of pressure and is consistent with Equation 3 of the main text.

S2 NOMENCLATURE OF KEY VARIABLES

	**Time-dependent variables**	
Q	nl/min	Flow rate as measured by the flow sensor
Q_s	nl/min	Flow rate into the system compliance
Q_ϕ	nl/min	Flow rate into the ocular compliance
Q_r	nl/min	Flow rate through the aqueous humour outflow pathway.
P	$mmHg$	Pressure as measured by the pressure sensor, representative of intraocular pressure
P_a	$mmHg$	Applied pressure
	**Pressure-dependent variables**	
ϕ	$nl/mmHg$	Ocular compliance
R	$mmHg/(\mu l/min)$	Hydrodynamic resistance of the outflow pathway
C	$nl/min/mmHg$	Conventional outflow facility ($C = 1/R$)
	**Variables for each step, j**	
Q_j	nl/min	Steady state flow rate for step j
P_j	$mmHg$	Steady state pressure for step j
$P_{a,j}$	$mmHg$	Applied pressure for step j
$P_{\phi,j}$	$mmHg$	Pressure corresponding to the measured compliance for step j using the Discrete Volume Method
ΔP_j	$mmHg$	Difference in steady state pressure P between two steps
$\Delta P_{a,j}$	$mmHg$	Difference in applied pressure P_a between two steps
ΔV_ϕ	nl	Change in intraocular volume for a change in intraocular pressure
ϕ_j	$nl/mmHg$	Ocular compliance for step j
λ_j	-	Non-linearity parameter arising in Step Response Method.
	**Variables that are constant for each eye**	
ϕ_s	$nl/mmHg$	System compliance
ϕ_r	$nl/mmHg$	Reference compliance at $P_{r,\phi}$
R_q	$mmHg/(\mu l/min)$	Combined hydrodynamic resistance of the flow sensor and capillary
R_c	$mmHg/(\mu l/min)$	Hydrodynamic resistance of the cannula
C_q	$nl/min/mmHg$	Hydrodynamic conductance of flow sensor and capillary
C_r	$nl/min/mmHg$	Reference facility at $P_{r,c}$
β	-	Non-linearity parameter characterising pressure dependence of outflow facility
γ	$mmHg$	Non-linearity parameter characterising deviation from Friedenwald's model
$P_{r,c}$	$mmHg$	Reference pressure for facility calculations
$P_{r,\phi}$	$mmHg$	Reference pressure for compliance calculations

S3 DETERMINING P_Φ FOR THE DISCRETE VOLUME METHOD

Ocular compliance changes as a function of pressure. Hence, ocular compliance measured over a pressure step by the Discrete Volume Method, $\Delta V_\phi/\Delta P$, corresponds to the true value of the ocular compliance at some pressure between P_{j-1} and P_j, which we term $P_{\phi,j}$. According to the mean value theorem, $\Delta V_\phi/\Delta P$ is equivalent to dV_ϕ/dP evaluated at $P_{\phi,j}$:

$$\phi\Big|_{P_{\phi,j}} = \frac{dV_\phi}{dP}\Big|_{P_{\phi,j}} \tag{S4}$$

Using Equation S3 to evaluate ϕ at $P_{\phi,j}$ and by applying the integral over the jth pressure step from P_j at $t=0$ to P_{j-1} at $t=T$, where $\Delta P_j = P_j - P_{j-1}$, we can write

$$\begin{aligned}
\phi_r\left(\frac{P_{r,\phi}+\gamma}{P_{\phi,j}+\gamma}\right) &= \frac{1}{\Delta P_j}\int_0^T Q_\phi\, dt \\
&= \frac{1}{\Delta P_j}\int_0^T \phi\frac{dP}{dt}\, dt \\
&= \frac{1}{\Delta P_j}\int_{P_{j-1}}^{P_j} \phi\, dP \\
&= \frac{1}{\Delta P_j}\int_{P_{j-1}}^{P_j} \phi_r\left(\frac{P_{r,\phi}+\gamma}{P+\gamma}\right) dP \\
&= \frac{\phi_r\left(P_{r,\phi}+\gamma\right)}{\Delta P_j}\ln\left(\frac{P_j+\gamma}{P_{j-1}+\gamma}\right)
\end{aligned} \tag{S5}$$

Cancelling $\phi_r\left(P_{r,\phi}+\gamma\right)$ from both sides yields

$$\frac{1}{P_{\phi,j}+\gamma} = \frac{1}{\Delta P_j}\ln\left(\frac{P_j+\gamma}{P_{j-1}+\gamma}\right) \tag{S6}$$

Hence

$$P_{\phi,j} = \frac{\Delta P_j}{\ln\left(1+\frac{\Delta P_j}{P_{j-1}+\gamma}\right)} - \gamma \tag{S7}$$

Using the Laurent series expansion $1/\ln(1+x) = 1/x + 1/2 - x/12 + \mathcal{O}\left(x^2\right)$ allows us to write an solution for $P_{\phi,j}$

$$P_{\phi,j} = P_j - \frac{\Delta P_j}{2}\left(1+\frac{1}{6}\left(\frac{\Delta P_j}{P_{j-1}+\gamma}\right)+\mathcal{O}\left(\frac{\Delta P_j}{P_{j-1}+\gamma}\right)^2\right) \tag{S8}$$

This reveals that to leading order, $P_{\phi,j}$ is simply the midpoint between P_{j-1} and P_j.

S4 ANALYSIS FOR THE STEP RESPONSE METHOD

In this section, we derive an analytical solution to the step response of a system with pressure-dependent resistance and compliance. We start with the simple lumped parameter model shown in Fig 1b, with R_c neglected andcompliances ϕ and ϕ_s in parallel . This yields the equation:

$$\frac{dP}{dt} + \frac{(C + C_q)P - C_q P_a}{\phi + \phi_s} = 0 \tag{S9}$$

where the compliance dependence on pressure is described by

$$\phi + \phi_s = \phi_r \left(\frac{P_{r,\phi} + \gamma}{P + \gamma} \right) + \phi_s \tag{S10}$$

The facility pressure dependence is given by

$$C = C_r \left(\frac{P}{P_{r,c}} \right)^{\beta} \tag{S11}$$

In the experiments, the applied pressure is adjusted in a series of steps and held constant until the pressure in the eye asymptotes to a steady value. For an arbitrary step starting at $t = 0$ the initial condition is defined as P_{j-1}, which is the steady state pressure of the previous step. The steady state asymptote for the jth step is found by setting the derivative in Equation S9 to zero which yields

$$P_j = \left(\frac{C_q}{C_j + C_q} \right) P_{a,j} \tag{S12}$$

where C_j is the facility at $P = P_j$. Because of the non-linearity due to the pressure dependence in both the compliance and facility, we seek a solution to Equation S9 using asymptotic analysis, where we assume that the size of the imposed pressure step, $\Delta P_j = P_j - P_{j-1}$, is small relative to the pressure at the end of the step, P_j.

We define the non-dimensional pressure p^* during step j according to

$$p^* = \frac{P_j - P}{\Delta P_j} \tag{S13}$$

which is equivalent to

$$P = P_j(1 - \varepsilon p^*) \tag{S14}$$

where

$$\varepsilon = \frac{\Delta P_j}{P_j} \ll 1 \tag{S15}$$

Substituting Equations S14 into Equation S9

$$-\varepsilon P_j \frac{dp^*}{dt} + \frac{(C + C_q)P_j(1 - \varepsilon p^*) - C_q P_{a,j}}{\phi + \phi_s} = 0$$

The pressure dependent facility (see Equation S11) can be written using a series expansion around $\varepsilon = 0$, to $\mathcal{O}(\varepsilon^2)$:

$$C = C_j \left(1 - \varepsilon\beta p^* + \frac{1}{2}\varepsilon^2\beta(\beta-1){p^*}^2\right) \tag{S16}$$

Substituting Equations S12 and S16 the differential equation becomes

$$\frac{dp^*}{dt} + \frac{\big((1+\beta)C_j + C_q\big)p^* - \frac{1}{2}\varepsilon\beta(\beta+1)C_j{p^*}^2}{\phi + \phi_s} = 0 \tag{S17}$$

Similarly the reciprocal of the pressure dependent total compliance can be written to $\mathcal{O}(\varepsilon)$:

$$\frac{1}{\phi + \phi_s} = \frac{1}{\phi_j + \phi_s}\left[1 - \frac{\varepsilon P_j \phi_j p^*}{(P_j + \gamma)(\phi_j + \phi_s)}\right]$$

where $\phi_j = \phi_r \left(\frac{P_{r,\phi}+\gamma}{P_j+\gamma}\right)$ is the ocular compliance at step j. Substituting this expression into S17 and collecting powers of ε, we obtain to $\mathcal{O}(\varepsilon)$

$$\frac{dp^*}{dt} + \frac{p^*}{\tau_j} - \frac{\varepsilon a_j {p^*}^2}{\tau_j} = 0 \tag{S18}$$

where

$$\frac{1}{\tau_j} = \frac{C_q + (1+\beta)C_j}{\phi_j + \phi_s}$$

and

$$a_j = \frac{\frac{1}{2}\beta(\beta+1)C_j}{C_q + (1+\beta)C_j} + \frac{P_j\phi_j}{(P_j+\gamma)(\phi_j+\phi_s)}$$

Equation S18 has the exact solution due to Bernoulli

$$p^*(t) = \frac{1}{k_j e^{\frac{t}{\tau}} + \varepsilon a_j}$$

where k_j is an arbitrary integration constant.

Using the initial condition $p^*(0) = 1$, yields $k_j = 1 - \varepsilon a_j$, hence:

$$p^*(t) = \frac{1}{(1-\varepsilon a_j)e^{\frac{t}{\tau_j}} + \varepsilon a_j} \tag{S19}$$

Substituting into Equation S13 and letting $\lambda_j = \varepsilon a_j$ we obtain the equation for $P(t)$

$$P(t) = P_j\left(1 - \frac{\Delta P_j}{P_j}\frac{1}{(1-\lambda_j)e^{t/\tau_j} + \lambda_j}\right) \tag{S20}$$

where using the definition of ε (Equation S15)

$$\lambda_j = \frac{\Delta P_j}{P_j} \left(\frac{1}{\phi_s + \phi_j} \right) \left[\left(\frac{\beta(\beta+1)C_j\tau_j}{2} \right) + \left(\frac{P_j\phi_j}{P_j + \gamma} \right) \right] \tag{S21}$$

and

$$\tau_j = \frac{\phi_j + \phi_s}{C_q + (1+\beta)C_j} \tag{S22}$$

Due to the dependence of λ_j on γ, an iterative process must be applied. The initial estimate of τ_j for this process can be calculated by omitting terms of $\mathcal{O}(\varepsilon)$, which is equivalent to setting $\lambda_j = 0$.
